# Isolated populations of the bush-cricket *Pholidoptera frivaldszkyi* (Orthoptera, Tettigoniidae) in Russia suggest a disjunct area of the species distribution

**DOI:** 10.3897/zookeys.665.12339

**Published:** 2017-04-04

**Authors:** Peter Kaňuch, Martina Dorková, Andrey P. Mikhailenko, Oleg A. Polumordvinov, Benjamín Jarčuška, Anton Krištín

**Affiliations:** 1 Institute of Forest Ecology, Slovak Academy of Sciences, Ľ. Štúra 2, 960 53 Zvolen, Slovakia; 2 Moscow State University, Department of Biology, Botanical Garden, Leninskie Gory 1, Moscow 119991, Russia; 3 Penza State University, Department of Zoology and Ecology, Lermontova 37, Penza 440602, Russia

**Keywords:** Declining species, Insecta, mountain habitats, mtDNA, phylogeography, range fragmentation

## Abstract

Phylogenetic analysis and assessment of the species status of mostly isolated populations of *Pholidoptera frivaldszkyi* in south-western Russia occurring far beyond the accepted area of the species distribution in the Carpathian-Balkan region were performed. Using the mitochondrial DNA cytochrome *c* oxidase subunit I gene fragment, we found a very low level of genetic diversity in these populations. Phylogeographic reconstruction did not support recent introduction events but rather historical range fragmentation. The grouping of the Russian and Romanian haplotypes in a distinct phylogenetic clade suggests that the pre-glacial range of *P. frivaldszkyi* had extended towards the Ponto-Caspian region, with considerable gene flow between different refugia. However, post-glacial northward expansion of the species from supposed Caucasus refugia contributed most likely to the current disjunct distribution of this relict-like bush-cricket.

## Introduction

The Green dark bush-cricket, *Pholidoptera frivaldszkyi* (Herman, 1871), is a regionally rare and endangered grassland-dwelling Orthoptera (Tettigoniidae) species with a decreasing population trend (Krištín and Iorgu 2014; Hochkirch et al. 2016). Genetic structure (mitochondrial DNA) and variation of morphological traits as individually specific pattern of black spots on the light green shield and head in fragmented and isolated populations of this flightless sedentary insect occurring in central and south-eastern Europe confirm the species’ relict-like character (Fabriciusová et al. 2008; Kaňuch et al. 2014). Scarce records have determined its main distribution area in the Carpathian Mountains and mountains on the Balkan Peninsula, with an emphasis on the former (e.g. Harz 1969; Nagy 2005; Warchałowska-Śliwa et al. 2005; Fabriciusová et al. 2008; Iorgu et al. 2008; Chobanov and Mihajlova 2010; Krištín et al. 2013; Hochkirch et al. 2016). Findings further to the north-east, beyond the Carpathian massif, reported from Ukraine and Russia were relatively old, unlocalised or incomplete (e.g. Medvedev 1954; Bey-Bienko 1964, 1970; Yakushenko et al. 1984; Heller et al. 1998). Such unreliable character of occurrence data together with questioning of the species status due to song differences in Russian populations (Heller 1988) gave rise to doubts and therefore the only distribution area of *P. frivaldszkyi* was validated for Carpathian-Balkan region also in the recent check-list of European Orthoptera fauna (cf. Hochkirch et al. 2016). However, several isolated populations have recently been found or rediscovered in Russia (Mikhailenko and Polumordvinov 2015) thus confirming the species presence in that area. Thanks to collected tissues of specimens we could perform phylogenetic analysis and assessment of the populations origin there as either a range fragmentation or the result of some introduction events. In this article we report the phylogeographic pattern of *P. frivaldszkyi* populations in Russia at the maternally-inherited mtDNA level and discuss our results with recent species inference from the Carpathian-Balkan region (Kaňuch et al. 2014).

## Materials and methods

**Figure 1.**
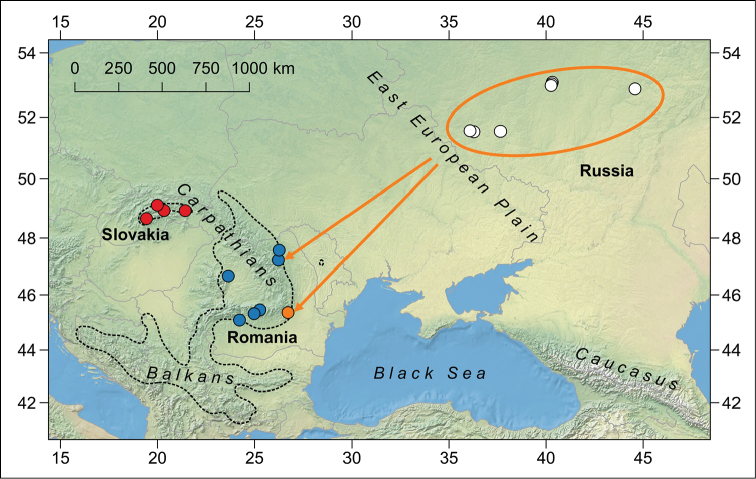
Sampled sites of *Pholidoptera frivaldszkyi* populations in Central and Eastern Europe. Three geographically homogeneous genetic clusters in the Carpathian Mountains defined by spatial analysis of molecular variance are colour coded according to Kaňuch et al. (2014). Arrows denote sites in eastern Romania where individuals that share the most similar haplotypes to populations found in Russia (ellipse) have occurred (see Fig. 2). The species range validated by the IUCN Red List (Hochkirch et al. 2016) is outlined by the dotted line.

A total of 26 adult individuals of *P. frivaldszkyi* (12 males and 14 females) were collected at seven sites (3–6 ind. per site) from the Kursk (sites Petropavlovka, Bogatyrevo, Panino), Tambov (Novospasskoe, Khobotovo, Ranino) and Penza (Krutec) regions in Russia (51.5367°–53.0925°N, 36.0906°–44.5840°E, 140–230 m a.s.l., Fig. 1; for a detailed description of the sites, see in Mikhailenko and Polumordvinov 2015), between June 30^th^ and August 3^rd^ 2016. Extraction of genomic DNA from muscle tissue and amplification of a fragment of the mitochondrial cytochrome *c* oxidase subunit I (*COI*) gene from each sampled individual followed the protocol described in Kaňuch et al. (2014). In the phylogenetic analyses, we employed the HKY+I model that was previously determined as the best-fit substitution site evolutionary model for the organism studied (Kaňuch et al. 2014). We reconstructed phylogenetic relationships among haplotypes using maximum-likelihood (ML) analysis and Bayesian inference. For construction of an ML tree we used the MEGA5.2 software (Tamura et al. 2011), for which an initial tree was built using the neighbour-joining method, and variants of the topology were created using the nearest neighbour interchange method. A bootstrap analysis with 1000 replicates was performed to determine the support values for each node. Bayesian inference was performed with MrBayes 3.2 software (Ronquist et al. 2012). Markov chain Monte Carlo sampling was performed with four chains that were run twice, producing one million replicates, and sampled every 100 generations, from which the first 250,000 generations were discarded as burn-in (subsequent combination of the runs was done in a default manner). The remaining trees were used to calculate the posterior probabilities of branches in a 50% majority-rule consensus topology. Sequences of four related *Pholidoptera* species and *Eupholidoptera danconai* were also amplified or downloaded from GenBank (https://www.ncbi.nlm.nih.gov/genbank/), respectively, in order to use them as outgroup species in rooted trees.

## Results and discussion

**Figure 2.**
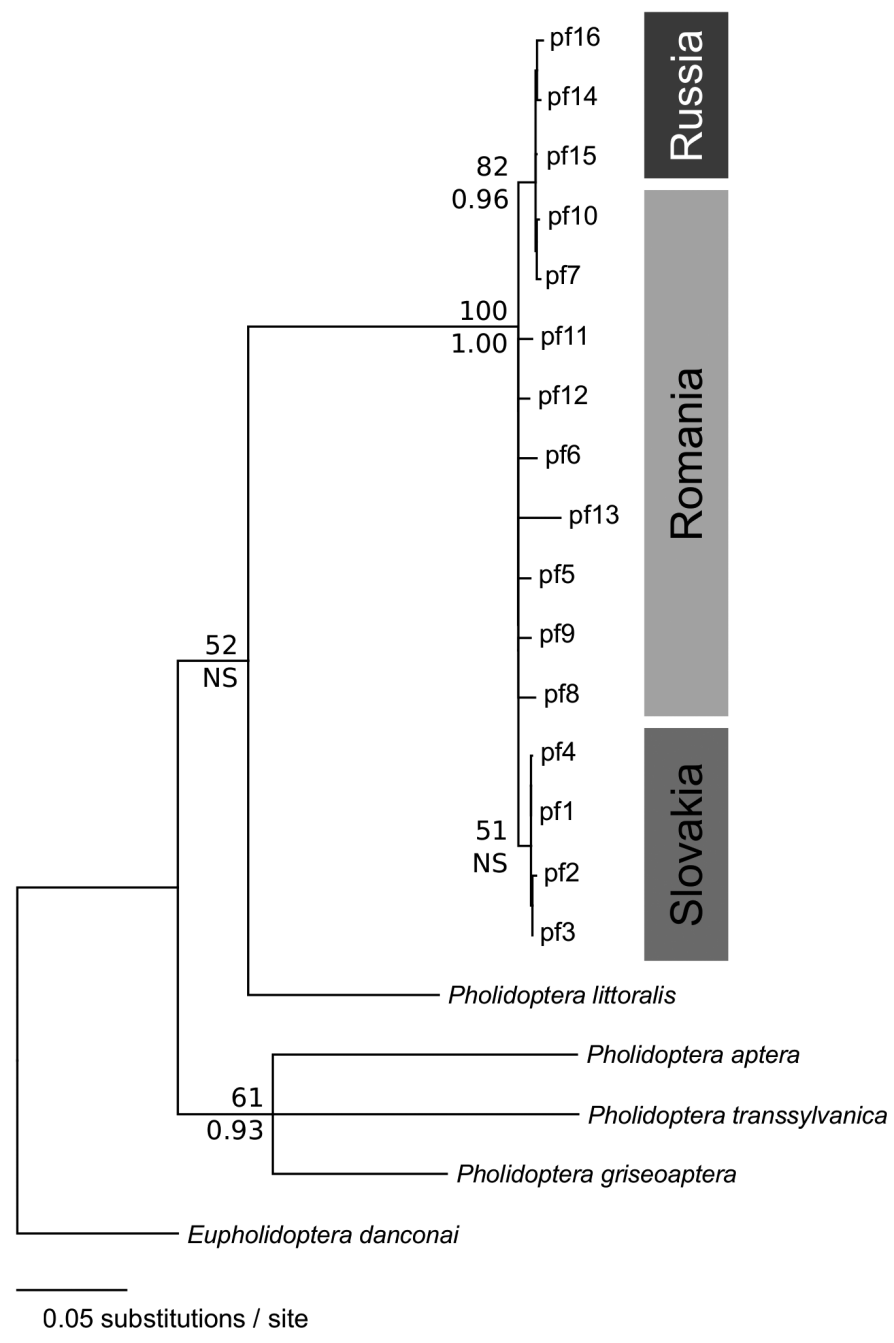
Maximum-likelihood (ML) phylogenetic tree for 16 haplotypes of *Pholidoptera frivaldszkyi* (pf1–pf16; GenBank accession numbers KF706416–KF706428, KY554960–KY554962) with outgroup species (KC852400, KY554963–KY554966) based on a 778 bp fragment of the mtDNA *COI* gene. Tree topology and branch lengths of Bayesian inference were congruent with ML analysis. Nodes with significant support values are indicated (upper, ML bootstrap > 50%; lower, Bayesian posterior probability > 0.90).

A very low level of genetic diversity was found within seven mostly isolated populations of *P. frivaldszkyi* in Russia. Analysis of the 778 bp long sequences of mtDNA *COI* gene in 26 individuals revealed only two (0.3%) variable sites with three unique haplotypes (GenBank accession numbers KY554960–KY554962), which may indicate a strong demographic decline (cf. Kaňuch et al. 2014). All but three individuals shared the haplotype pf14 (haplotype pf15, 2 ind., Bogatyrevo; pf16, 1 ind., Panino). The Russian and two other haplotypes from eastern Romania (pf7, pf10) grouped together in a distinct clade having significant statistical support in both phylogenetic reconstructions (Fig. 2). Furthermore, the high level of genetic differentiation in eastern Romania previously found by spatial analysis of molecular variance (Kaňuch et al. 2014) suggests a possible phylogeographic connection between the Russian and Carpathian populations (Fig. 1). The ratio of the black spotted area on the males’ shields, a quantitative trait that retains useful information on population genetic structure (Kaňuch et al. 2014), has also supported such a scenario. In Russian specimens, the light or intermediate phenotype prevailed (data not presented), similarly to the Romanian specimens (Kaňuch et al. 2014).

The well preserved mountain meadows in the Carpathian-Balkan region (average altitude 650 m a.s.l.) are considered as typical habitat for *P. frivaldszkyi* (e.g. Nagy 2005; Fabriciusová et al. 2008; Iorgu et al. 2008; Krištín et al. 2013); thus, one could have expected that lowland populations in the remote (1300–2400 km) East European Plain should be founded by unintentional vector-born or human-mediated introductions. Similar events have already been confirmed in some Orthoptera species (Wagner 2004; Kaňuch et al. 2013). However, the almost uniform genetic diversity of the mostly isolated Russian populations spread over a relatively large area (similar in size to the populations in the Carpathians; Fig. 1) rejects this hypothesis. A reverse option that some individual lineages in eastern Romania were introduced recently from Russia is more likely. However, differentiation among and within all the Romanian populations is less pronounced compared to adjacent (~500 km) and genetically and morphologically well differentiated cluster of populations in Slovakia (cf. Kaňuch et al. 2014).

The most plausible evolutionary explanation of the observed phylogeographic pattern is range contraction during the last glacial period and subsequent post-glacial species expansion. Traces of a genetic connection between the eastern Romanian and Russian populations suggest that the pre-glacial range of *P. frivaldszkyi* also extended towards the Ponto-Caspian region, similarly to the ranges of many other recent Orthoptera species (e.g. Heller et al. 1998). After the retreating of ice (<18 ka before the present), re-colonisation of Eastern Europe and Russia would then have been from the southern Carpathians or Balkans and from the Caucasus refugia (Hewitt 1999). Such post-glacial expansions of some eastern lineages from the Caucasus towards the north have been also inferred in similar relict (Todisco et al. 2010) and related insect species (Cooper et al. 1995). On the other hand, the little genetic differentiation found between these lineages (Fig. 2) is probably due to considerable gene flow between the Mediterranean and Pontic refugia during the last ice-age (Schmitt 2007). Although some very isolated populations in the East European Plain (see Bey-Bienko 1964; Pushkar 2009) as former stepping stones between the Carpathian-Balkan and Russian lineages may still exist (cf. Chobanov and Mihajlova 2010), the gene flow would be very restricted nowadays (Habel et al. 2010). Thus, strong historical range fragmentation most likely produced the current disjunct distribution of *P. frivaldszkyi*. This situation could therefore attract even more attention on the evolution and ecology (e.g. relict habitats, Kaňuch et al. 2014, Mikhailenko and Polumordvinov 2015; song differences, Heller 1988) of this bush-cricket.
